# High-cytocompatible semi-IPN bio-ink with wide molecular weight distribution for extrusion 3D bioprinting

**DOI:** 10.1038/s41598-022-10338-1

**Published:** 2022-04-15

**Authors:** Meiqi Li, Tingchun Shi, Danyu Yao, Xiuyan Yue, Haoxuan Wang, Kezhou Liu

**Affiliations:** 1 School of Automation, Hangzhou Dianzi University, Hangzhou, China; 2 Engineering for Life Group (EFL), Suzhou, China; 3 Library of Hangzhou Dianzi University, Hangzhou, China

**Keywords:** Tissue engineering, Biomedical engineering, Biomaterials - cells, Biomedical materials, Tissues

## Abstract

The development of 3D printing has recently attracted significant attention on constructing complex three-dimensional physiological microenvironments. However, it is very challenging to provide a bio-ink with cell-harmless and high mold accuracy during extrusion in 3D printing. To overcome this issue, a technique improving the shear-thinning performance of semi-IPN bio-ink, which is universally applicable to all alginate/gelatin-based materials, was developed. Semi-IPN bio-ink prepared by cyclic heating–cooling treatment in this study can reduce the cell damage without sacrificing the accuracy of the scaffolds for its excellent shear-thinning performance. A more than 15% increase in post-printing Cell viability verified the feasibility of the strategy. Moreover, the bio-ink with low molecular weight and wide molecular weight distribution also promoted a uniform cell distribution and cell proliferation in clusters. Overall, this strategy revealed the effects of molecular parameters of semi-IPN bio-inks on printing performance, and the cell activity was studied and it could be widely applicable to construct the simulated extracellular matrix with various bio-inks.

## Introduction

3D bioprinting can precisely control the deposition of cells and materials, and produce bioactive tissue scaffolds, which has been widely concerned in recent years^1–3^. At present, the additive manufacturing technologies mainly used in tissue engineering include extrusion printing^4^, ink-jet printing^5,6^ digital light-curing printing, and laser printing^5,7–14^. Extrusion printing technology^15,16^ has been widely used in the field of biological manufacturing due to its multiple printing platforms, easy operation, and wide range of printing ink^14,17^.

Since extrusion printing has had the lowest observed cell viabilities, it has the largest platform of work investigating methods to increase viability while maintaining high printing resolution^18^. Currently, the main research is to print cell-loaded scaffolds by changing the size of the nozzle inner diameter and the ratio of bio-ink^19^. If the material viscosity is high, the shear stress can be reduced by increasing the inner diameter of the nozzle, so that the material is extruded with reducing the cell damage^17,20^. The disadvantage is that the forming accuracy decreases^20^. Without changing the size of the inner diameter of the nozzle, the high-viscosity bio-ink has a good molding effect. However, the disadvantage is that the high shear force reduces the cell activity. To balance higher printability and cell viability, previous reports evaluated the printability of sodium alginate-gelatin gels with different concentrations. However, the principle of tissue engineering to fabricate biomimetic scaffolds is to mimic the composition and structure of natural tissues as much as possible. The material with high viscosity has a good molding effect, but it is not conducive to cell growth and low viscosity can be printed poorly. The preparation of extruded 3D printing bio-ink with the same concentration, ratio of materials, and the nozzle inner diameter without reducing printability and cell activity has not been studied. Therefore, the design of a bio-ink with adjustable rheological properties and no changing the material ratio and concentration is of great significance to promote the extrusion 3D bio-printing technology in tissue engineering and clinical application.

The factors affecting the properties of printing ink flow are the molecular weight and distribution of materials, the structure of molecular chains, and the external factors including temperature and external forces^17,20^. Higher molecular weight inks tend to have longer chains and more intermolecular entanglement, which impedes the fluidity of the material, that is, the material has a greater viscosity^21^. The shear sensitivity of materials with wide molecular weight distribution is also stronger. In addition, the branched-chain length of the molecule is positively correlated with the viscosity. According to the Arrhenius equation, the flexibility of molecular chains is related to temperature, shear rate, and shear stress^22^. A polymer with a harder molecular chain has a greater intermolecular force and activation energy for viscous flow, so its viscosity is more sensitive to temperature^23^. The polymer has a higher softness of the molecular chain and a larger flow activation energy, and the viscosity of the material is more sensitive to the shear rate^24^. Because the flexible molecules of polymer with is more easily changed with shear rate and the viscosity is changed more with shear rate^21,25–27^. Therefore, the structure of the molecular layer is essential for extruded 3D printing bio-ink.

Many studies have used 3D printing to prepare hydrogel scaffolds to simulate natural Extracellular matrix (ECM). ECM is a 3D microenvironment surrounding cells, which is essential to the biological tissue and organ function^28^. It is mainly composed of water, protein, collagen elastin fibronectin laminin, and polysaccharide^29^. ECM can provide support for structural integrity and flexibility and can be passed adhered to regulate cell migration Proliferation, differentiation, and survival of biochemical and biological signals^28,30^. Composite hydrogel composed of gelatin and sodium alginate is one of the basic materials for extrusion printing to mimics the extracellular matrix^31^. First, gelatin is a derivative of collagen, and sodium alginate is a polysaccharide^31–33^, similar to the composition of natural ECM. Secondly, gelatin has appropriate rheological properties and temperature sensitivity, and sodium alginate crosslinked by calcium ions can make up for its poor mechanical properties. They form a stable and high biocompatibility semi-IPN hydrogel composed of a crosslinked hydrogel and an uncrosslinked hydrogel, which is conducive to 3D extrusion printing^32,34,35^. Furthermore, uncrosslinked hydrogels in semi-interpenetrating hydrogels slowly dissolve after a period of time to provide space for cell proliferation and diffusion, while crosslinked polymers can provide mechanical support^28,36^. Therefore, gelatin and sodium alginate were used as bio-inks to improve cell activity during the extrusion printing process.

In this study, a high shear sensitivity semi-IPN bio-ink (sodium alginate and gelatin) suitable for extrusion-based 3D printing was developed, which improves the activity of cells without affecting the printing accuracy. Adjustable shear-sensitive bio-inks were prepared using the same concentration of gelatin/sodium alginate by controlling the number of cycles of heating–cooling to change the structure at the molecular level of the material. Firstly, FTIR and TGA were used to test and analyze changes in material properties; Secondly, the GPC and rheological properties of three SA/G bio-inks were characterized, and the relationship between the molecular weight and shear-thinning was got by their results. Then, the internal interconnected pore structure and mechanical properties were studied. Cell viability in the printed scaffolds was counted. It is verified that the preparation of semi-IPN bio-ink with high shear sensitivity suitable for extrusion printing with the same concentration and ratio of materials and no change about the nozzle can improve the cell survival rate post-printing and cell proliferation in clumps after culture.

## Materials and methods

### Materials

Gelatin (type A, 90–110 bloom derived from porcine skin) were purchased from Sigma Aldrich (USA); Sodium alginate (Aladdin, China); Nanoscale hydroxyapatite (Aladdin, China); Micron hydroxyapatite (Alighting, China); Anhydrous calcium chloride (McLean, China).

### Preparation method of SA/G bio-ink

Bio-ink formulations were prepared with the same ratios of alginate and gelatin and different heating–cooling times. Briefly, gelatin was dissolved in 20 mL of deionized water on a rotational shaker at 37 °C for 60 min until completely dissolved to obtain 20 wt% gelatin solution. Sodium alginate and 20 mL of deionized water were added to the gelatin solution described above and mixed and heated at 80 °C for 1 h. The gelatin concentration of the solution was 10% (w/v) sodium alginate and 2.5% (w/v) sodium alginate as samples of SA/G-A bio-ink.

Afterward, the preparation method of SA/G-B was that SA/G-A bio-ink was heated at 80 °C for 1 h and then cooled to room temperature. SA/G-B was heated at 80 °C for 1 h and cooled to room temperature to prepare SA/G-C bio-ink. These samples are called SA/GA, SA/G-B, and SA/G-C. The preparation method is shown in Table 1.

**Table 1:** Material composition and preparation methods of samples.

Hydrogel	SA/G-A	SA/G-B	SA/G-C
Sodium alginate:gelatin	4 g:1 g	4 g:1 g	4 g:1 g
Repeated heating/cooling (times)	1	2	3

### Cell extraction and culture

An 8-week-old SD rat was purchased from the experimental animal center of Zhejiang province. All methods were carried out by relevant guidelines and regulations. This study was carried out in compliance with the AVME guidelines. The SD rat had body weights of 207.4 ± 0.3 g from which chondrocytes were extracted. The rats euthanized by intraperitoneal injection of 2% penbital sodium solution according to the standard of 45 mg/kg were immersed in 75% alcohol for 10 min and then put into a clean bench. After the skin and soft tissue of the leg were cut, the femur was dislocated. The femoral head, femoral condyle, and tibial plateau were separated and washed with sterile PBS. The cartilage samples were incubated with type II collagenase sterilized by a 10 mL filter (0.22 μm filter, Jintang, China). The collagenase solution was removed and then the cartilage specimens were washed with sterile phosphate buffer 3 times. Next, the cartilage was digested with 10 mL of trypsin EDTA for 4 h. Then the cells were centrifuged at 1000 rpm for 5 min at room temperature and then blown with a 2 mL culture medium for 1 min. The cells were cultured in Dulbecco’s modified Eagle s medium (DMEM-F12) supplemented with 1 vol% penicillin/ streptomycin and 10 vol% fetal calf serum at 37 °C and in a humidified atmosphere with 5% CO_2_. The fresh medium was changed twice a week. After 80% fusion, the cells were passaged. When the confluence reached 80–90%, the cells were separated with 2 mL of trypsin EDTA, and the centrifuged cells were resuspended and mixed with bio-ink. All procedures were conducted in compliance with the ARRIVE guidelines, approved by institutional ethical review committees (Ethics Committee of Zhejiang University), and conducted under the authority of the Project Licence (12511).

### 3D bioprinting

**Figure 1:**
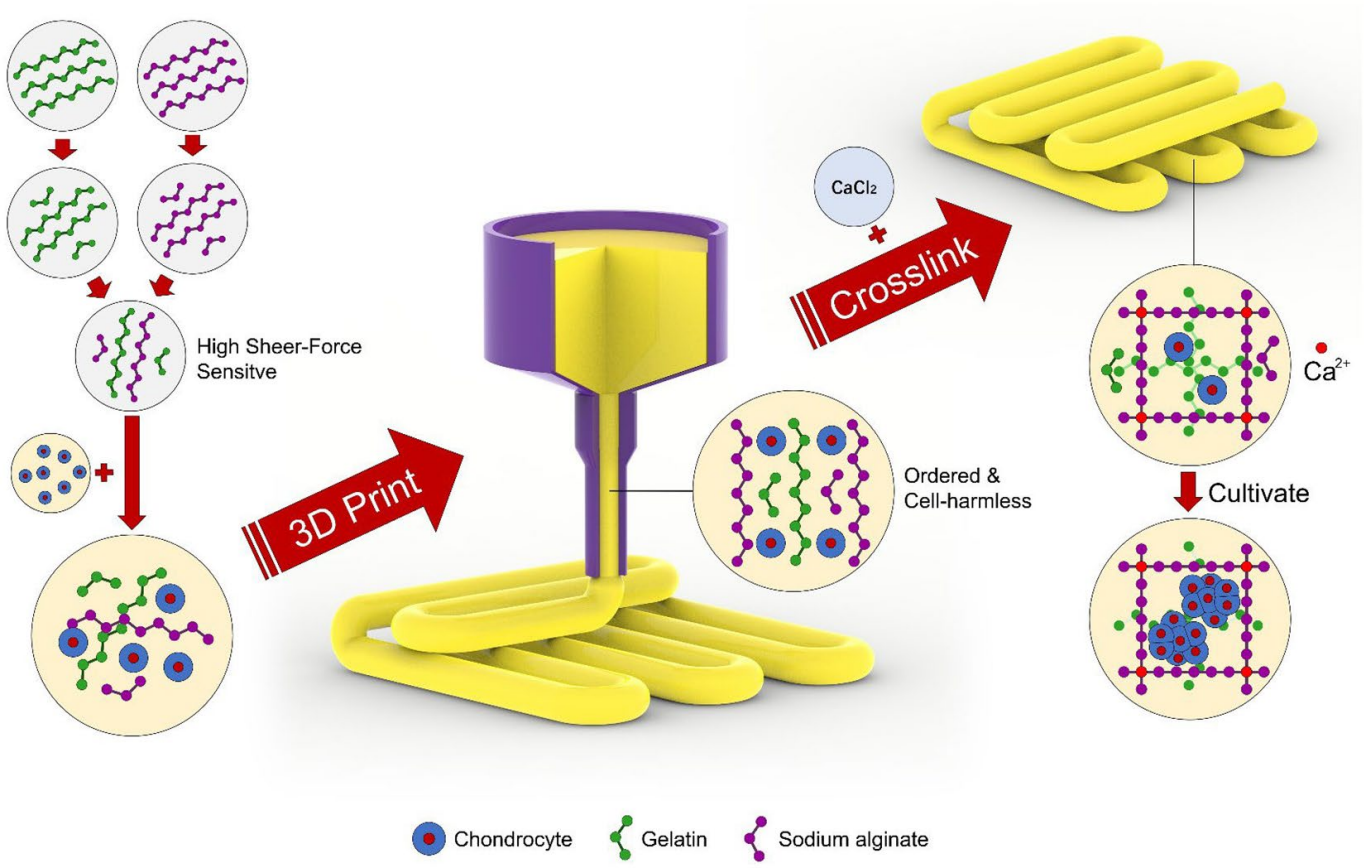
Strategy capable of generating highly bioactivity and precision structures.

This study used a CCA-II cell-controlled assembly printer. Three-dimensional modeling software SolidWorks and computer-aided manufacturing (CAD) technology were used with X–Y–Z motion platform, heater-free nozzle (200 um), cooling system (− 20 to 30 °C), and temperature controller (Fig. 1). A cube of 10 mm length, 10 mm width, and 5 mm height were designed with Solidworks software. The fibers were deposited layer by layer according to the design outline and path. SA/G-A, SA-B, and SA/G-C were printed at 25.5 °C, 23.5 °C, and 21.5 °C, respectively. Sterile high-precision blunt needles (Lindane, China) of specification (25G, inner diameter 0.25 mm) were fixed to a 1 mL plastic cartridge for printing, and 1 mL biological inks were injected into each cartridge. After printing, the scaffolds were immersed in 4 wt% CaCl_2_ solution for 1 min.

The pre-hydrogels were centrifuged at 5000 rpm for 1 min to remove the trapped bubbles and their concentration was twice that of the working hydrogels. Then, the chondrocytes were mixed with pre-bio-inks at a 1:1 ratio, and the cell concentration was 1 × 10^6^cells/ml. All scaffolds were prepared by the printing process described as above printing, keeping them sterile during the whole process of cell printing. The bioprinted samples were then kept in 12-well plates containing cell culture media and incubated at 37 °C.

### ATR-FTIR analysis

The freeze-dried SA/G-A, SA/G-B, and SA/G-C hydrogels and SA/G-AC, SA/G-BC, and SA/G-CC hydrogels crosslinked by 4% calcium chloride solution were evaluated by attenuated total reflection Fourier transform infrared spectroscopy (ATR-FTIR) technique (Bruker Equinox FTIR spectroscopy, Germany). The measurements were carried out at room temperature with wavenumbers ranging from 400 to 4000 cm^−1^.

### Thermogravimetric analysis

The thermogravimetric test was completed using a thermogravimetric analyzer. It is heated in air at a heating rate of 10 °C/min, ranging from room temperature to 400 °C.

### Gel permeation chromatography Determination of molecular weight

The molecular weight and distribution of SA/G-A, SA/G-B, and SA/G-C hydrogels were determined by gel permeation chromatography (GPC). The molecular weight and distribution of the hydrogels were determined by series connection of GPC Agilent 1100 and hydro super hydrogels M120, TM250, and TM500 (7.5 × 300 mm). 0.002 wt% of the water sample solution was filtered through 0.4 μm pore membrane before injection to remove dust particles. The flow rate of the mobile phase (sodium nitrate 0.1 m) was 1 mL/min. Before measurement, the equipment was calibrated using PEG standardized preparation equipment.

### Rheological evaluations

The viscoelastic properties of the SA/G samples were tested with the MCR301 rheometer (Antonpal, Austria). A parallel-plate geometry (25 mm diameter) with 0.5 mm gap distance was used for rheological assessments. Shear rate sweep (0.01–200 s^−1^) was evaluated to measure storage (G′) and loss (G″) moduli to characterize shear thinning behavior of the materials in a linear strain region of 0.1%, and SA/G-A, SA/G-B, and SA/G-C hydrogels were respectively performed at 25.5, 23.5, and 21.5 °C. Shear rate sweep (0.01–200 s^−1^) was evaluated to measure storage (G′) and loss (G″) moduli to characterize the shear-thinning behavior of the materials in a linear strain region of 0.1%.

Rotational recovery measurements were performed to characterize the materials recovery behavior by applying a low shear rate 200 s^−1^ for the 60 s, followed by a high shear rate at 0.01 s^−1^ for 60 s and 4 cycles. SA/G-A, SA/G-B, and SA/G-C hydrogels in recovery and shear rate sweep measurements were respectively performed at 25.5, 23.5, and 21.5 °C.

### Scanning electron microscope (SEM)

The scaffolds were then flash frozen in liquid nitrogen, exposing the cross-section of the sample. Platinum was plated on the surface and cross section of freeze-dried scaffolds by sputtering. Finally, the SEM images were taken by Hitachi s-4800 microscope at an acceleration voltage of 15 kV.

### Mechanical property test

The compression test was carried out with WDX-100 electronic universal testing machine (Zhejiang Wenzhou Weidu Electronics Co., LTD.), the compression strain rate was 1.0 mm/min, and the compression young's modulus was determined by the slope of the elastic region of the stress–strain curve. The sample is square (10 mm long, 5 mm thick). A total of 5 specimens were tested.

### In vitro studies

Cell viability of the scaffolds at days 1, 4, 7 were conducted using a live/dead viability kit (LIVE/DEAD Viability/Cytotoxicity Kit, Yeasen, China). The assay dye was prepared by mixing PI (4.5 μM) and Calcein-AM (6 μM) in PBS. The printed cell encapsulated chondrocytes scaffolds were washed with PBS, incubated for 30 min in the live/dead dye solution, and visualized by a confocal laser scanning microscope (Olympus FV 3000, USA). Four representative images were taken from different areas of a single construct. For each time point, samples were analyzed in triplicate. The live and dead cells at the different times points (1, 4 and 7 days) were counted from the images by Image J software (NIH) and compared.

Cell adhesion, spreading, and proliferation were evaluated through fluorescent staining of F-actin filaments with TRITC phalloidin (Yeasen, China) and cell nuclei with DAPI (Solarbio, China). Briefly, the cell-loaded scaffolds were fixed in 4% (w/v) paraformaldehyde (Solarbio) for 20 min and then permeabilized with 0.1% (w/v) Triton X-100 solution in 1xPBS (Procell, China) for 5 min. Next, samples were then incubated with TRITC phalloidin (1:200 dilution in 0.1% BSA) at room temperature for 30 min. After three times washing with 1xPBS, the samples were counterstained with DAPI for 20 min followed by washing three times with 1xPBS^37^. fluorescent images were acquired using a confocal laser scanning microscope (Olympus FV 3000, USA).

### Statistical analysis

All experiments were repeated in triplicate independently, and the data were expressed as mean ± standard deviation. Statistical analyses were performed using a two-tailed Student’s *t* test with *p* < 0.05 as the criterion for statistical significance.

### Ethical statement

All methods were carried out following relevant guidelines and regulations. All procedures were conducted in compliance with the ARRIVE Guidelines and AVMA Guidelines, approved by institutional ethical review committees (Ethics Committee of Zhejiang University), and conducted under the authority of the Project Licence (12511).

## Results and discussion

### FTIR analysis of bio-inks after repeated heating/cooling circularly

**Figure 2:**
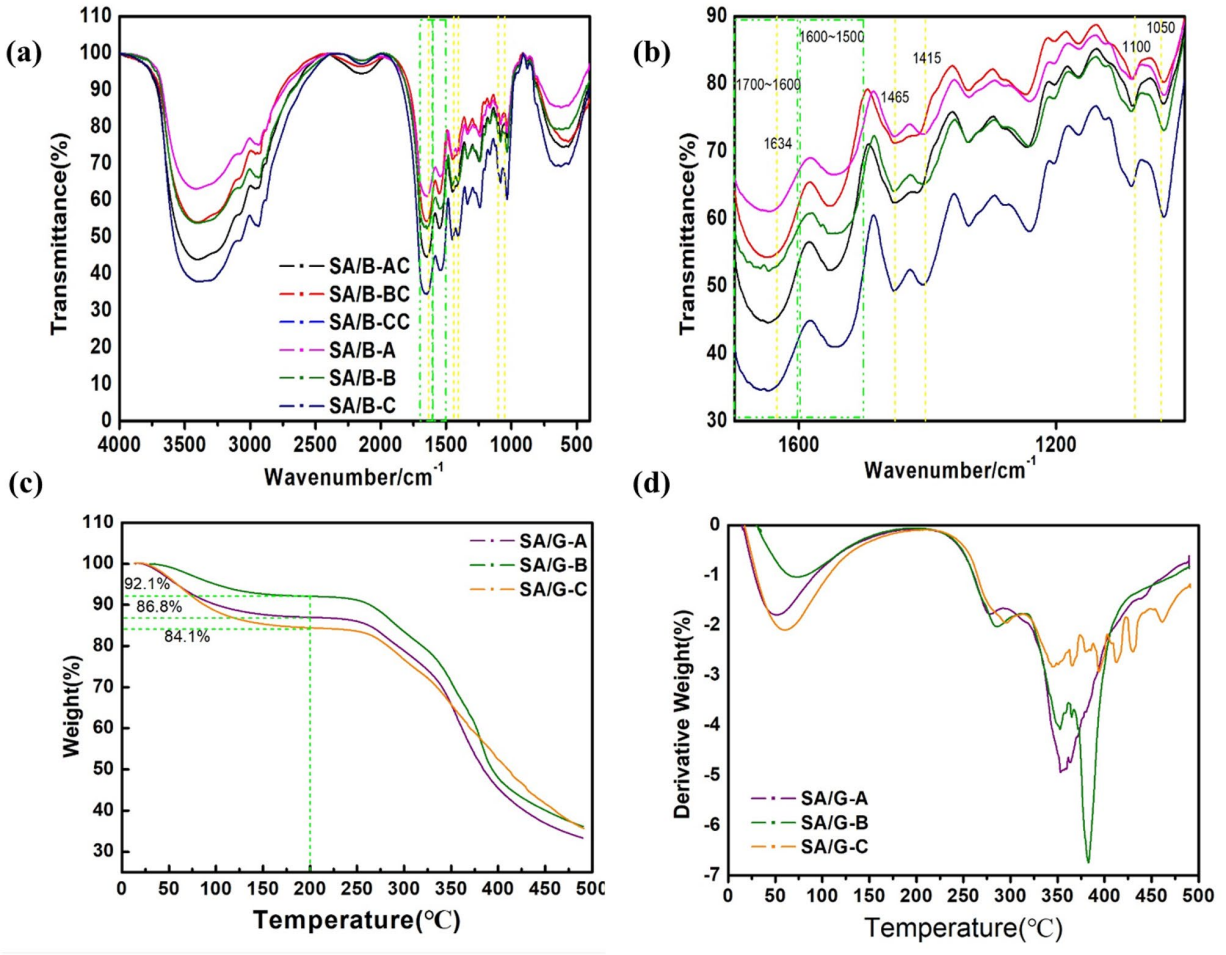
FTIR property and thermal property. (**a**, **b**) FTIR spectra; (**c**) TGA curves and (**d**) DTG curves.

Figure 2a, b shows the FTIR spectra in the percentage of transmittance for different concentrations of alginate-gelatin bio-ink over a spectral of 4000–200 cm^−1^. Here, peak shifts, differences in peak shapes, and the appearance of new bands can be observed dependent on changes in the hydrogel composition^38^. These main differences between SA/G hydrogels crosslinked with Ca^2+^ and SA/G hydrogels uncrosslinked reveal four peaks appearing at 1465 cm^−1^, 1415 cm^−1^, 1100 cm^−1^ and 1050 cm^−1^. The first two peaks at 1465 cm^−1^ and 1415 cm^−1^ are proposed to be the asymmetric and symmetric stretch vibration of –COO^−^ associated with carboxylic acid salts^39^ and are specific to the ionic bonding. Moreover, the peaks around at 1100 cm^−1^ relating to the C–C and C–O stretching, which considerably strengthens in the spectrum of SA/G bio-inks with Ca^2+^ ions, can be also attributed to the presence of crosslinking. The peak of C–C stretching (around 1050 cm^−1^) shows a higher intensity, suggesting either a stronger O–H binding vibration or a stronger binding of the Ca^2+^ to the guluronic acids of alginate. There were no significant differences in other absorption bands between six different hydrogels. The characteristic absorption band over 3000 cm^−1^ indicated the region of hydrogen-bonded –OH stretching or hydroxyl group^40^. The absorption band at 1634 cm^−1^ suggested that there was an interaction between negative charges of –COO^-^ groups in sodium alginate with the positive charges of –CONH_2_ in gelatin^41^. The two main absorption bands lying between 1700–1600 cm^−1^ and 1600–1500 cm^−1^ were attributed to Amide I and Amide II, respectively. The Amide I band is attributed to C=O stretching vibrations of the peptide bonds, that were regulated by the secondary structure of protein such as the α-helix and β-sheet^42^. The Amide II band is associated with the C–N stretching vibrations and N–H bending of the amino acid. All samples modified by the repeated heating–cooling treatment showed no changes in wavenumbers for amide I and amide II peak positions indicating an intact primary protein structure of gelatin^40,43^. Through heat-cool treatment, intramolecular hydrogen bonds are broken and replaced by hydrogen bonds to water molecules, thus preventing reconstruction of the inherent tertiary protein conformation^43,44^.

Nevertheless, FTIR results showed that the primary structure of gelatin was intact and unaffected by dissolution temperature, allowing alginate cross-linking as previously described^43,45^. Thus, the cyclic heating treatment did not destroy the main functional groups of gelatin and sodium alginate. In other words, the bio-inks were synthesized to contain the same proteins and polysaccharides to support the growth of the cells.

### Thermal stability

TGA thermograms of SA/G-A, SA/G-B, and SA/G-C are shown in Fig. 2c, d.

The decomposition of SA/B-C is the fast in 0–200 °C (Fig. 2c) and then it started to degrade at a steady rate compared to the others over 200 °C (Fig. 2d). Nevertheless, their fast decomposition temperature of SA/G-A, SA/G-B, and SA/G-C all were 200 °C. This may be because the broken molecular chain decomposes most at 200 °C, but the long chain is rarely destroyed^31^. When the temperature rises again, the remaining long molecular chains are destroyed^31^. The number of short molecular chains in SA/G-C is the largest, so its initial degradation is very fast, and the final residual amount is almost the same^31,46,47^. Thus, these results indicated that the thermal stability of gelatin/sodium alginate was obviously changed by cyclic heating–cooling treatment.

### Molecular weight and its distribution

**Figure 3:**
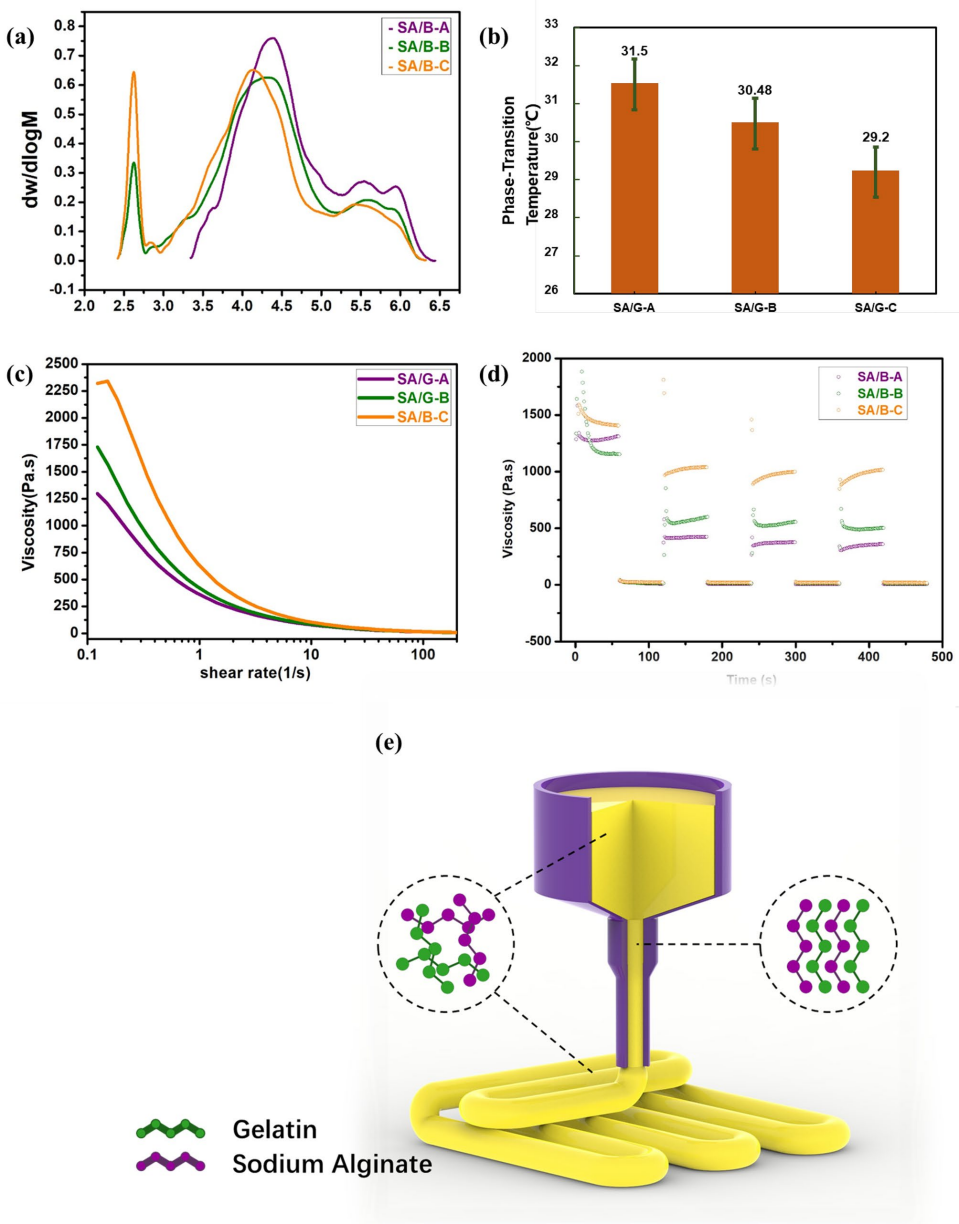
Molecular weight and rheological characterization of bio-inks. (**a**) The molecular weight distribution of SA/G-A, SA/G-B, and SA/G-C; (**b**) Phase transition temperature of gel-sol; (**c**) Viscosity-shear rate diagram; (**d**) The thixotropy of bio-inks and (**e**) Schematic diagram of shear-thinning mechanism.

Figure 3a and Table 2 show the molecular weight and distribution of the three bio-inks. It can be seen from Fig. 3a that the molecular peak moved to the left and the distribution of copolymer molecular weight became broad with the increase of heating–cooling times. With the increase of heating and cooling times, the probability of molecular chain breakage of hydrogels increases^48^. The molecular weight of the polymer has polydispersity, so the amorphous polymer has no clear viscous flow temperature, but a wide softening region^49^. In this area, the polymer is easy to flow, which is conducive to processing and molding^50^.

**Table 2:** GPC results of samples.

Name	Mn	Mw	Mw/Mn	Unit	Area,%
SA/G-A	17,149	140,128	8.17	Da	100
SA/G-B	2833	86,483	30.527	Da	100
SA/G-C	1841	69,701	37.86	Da	100

It can be seen from Table 2 that the Mw/Mn ratio of SA/G-C is the largest. That is to say, it has the largest dispersion and the widest molecular weight distribution. Previous studies have shown that the wider the molecular weight distribution is, the more sensitive the material is to shear stress^26^. At a low shear rate, long molecular chains with wide distribution are easy to entangle^50^. At this time, the wider the molecular weight distribution, the higher the viscosity; At a high shear rate, the entangled long molecular chain is easy to be destroyed. At this time, the wider the molecular weight distribution is, the smaller the viscosity is^25,27^. Thus, when bio-ink is extruded, the cells in SA/G-C may experience the least shear force.

### Rheological properties

The phase transition temperatures of SA/G-A, SA/G-B, and SA/G-C are shown in Fig. 3b. The transition temperature is the temperature of the sol–gel transition, which is determined by the cross storage modulus (G′) and the loss modulus (G″). The phase transition temperatures of SA/G-A, SA/G-B, and SA/G-C are 31.5 °C, 30.48 °C and 29.2 °C, respectively. This may be related to the change of molecular structure caused by heating treatment^21,51^. Therefore, data on phase transition temperatures also confirm heating–cooling treatment may make the molecular chain of the material shorter.

Shear-thinning is the principle of SA/G bio-ink extrusion molding^25,46^. As shown in Fig. 3e, the microscopic explanation of shear-thinning is that when the shear effect is stronger than Brownian motion, the entangled molecular chains tend to stretch, deform, disperse, and be ordered. The shear-thinning behavior is generally reversible. When the shear force disappears, the molecular aggregates are reformed due to its Brownian motion, that is, the chain-like colloidal molecules return to their natural position of non-orientation and return to the entangled state^21,25^. Figure 3c shows the shear-thinning characteristics of bio-inks. It can be seen from Fig. 3c that the slope of SA/G-C’s curve is the smallest, followed by SA/G-B, and SA/G-A is the smallest. In other words, the shear sensitivity of these three bio-inks is SA/G-A<SA/G-B<SA/G-C. The rheological data verified the previous guesses in sections "Thermal stability" and "Molecular weight and its distribution". The recovery behavior of hydrogel inks was especially important for the post-printing behavior of each printed scaffold^17^. Physically, recovery allows the bio-ink to rapidly increase in viscosity after extrusion and to maintain a high shape fidelity^17,43^. All samples show excellent recovery to their initial viscosity over the 180–480 s periods, demonstrating all changes in polymer structure or properties as a result of exposure to the high bioprinting shear conditions over the 180 s periods^17,25,35,46^. SA/G-C showed the fastest recoveries after application of the high shear rate compared to the SA/G-A as well as SA/G-B at first 180 s. This could also be attributed to wider molecular weight distribution in comparison to SA/G-A as well as SA/G-B^17,25,35,46^. Thus, it is suitable for semi-IPN with more molecular weight distribution to be printed.

When the extrusion molding effect is the same, the shear force of the bio-ink with high shear sensitivity is smaller and the damage degree of cells is the smallest^17,25,35,46^. When the shear force disappears, the curing speed of high shear sensitivity biological ink is faster and the molding precision is high^21,25,46^.

Figure 3 revealed the relationship between molecular structure and rheological properties and hinted at their effect on cells suffered extrusion. The rheological properties showed that the shear sensitivity of the materials is controlled by the number of heating–cooling cycles. The rheological behavior of bio-ink is generally related to molecular weight and distribution, molecular chain structure, and softness^21,25–27^. The cells in bio-ink with high shear sensitivity suffered less damage during printing.

### Scaffolds morphologies

**Figure 4:**
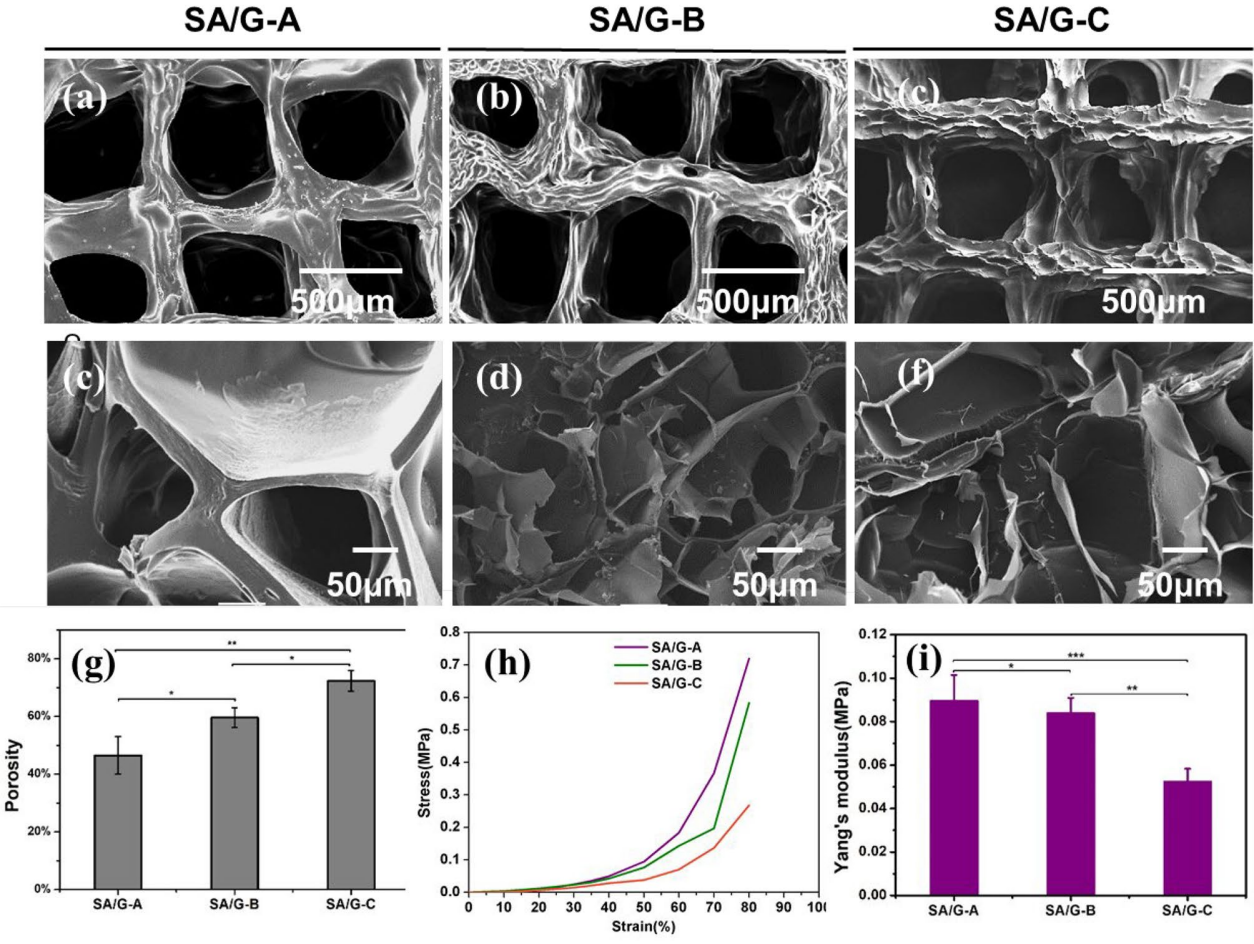
Physical properties of scaffolds. (**a**–**f**) Photographs taken with SEM showed multi-scale pore size, (**g**) Porosity of scaffolds, (**h**) compressive stress–strain curve, and (**i**) Young’s Modulus of printed scaffolds measured after crosslinking by CaCl_2_. All data are displayed as mean ± SD. ***indicates *p* < 0.001, **indicates *p* < 0.01, *indicates *p* < 0.05, analysed via Welch's *t* test.

SEM analysis revealed the influence of molecular structure on microstructure morphologies of freeze-dried hydrogels after printing. Figure 4a–c displayed printed macropore structure of scaffolds, which represented similar print accuracy. The printability of hydrogels was not weaken by the preparation method of cyclic heating preparation. Cross-section of Scaffolds (Fig. 4d–f) showed a continuous and porous structure by virtue of the freeze-drying step, resembling other macromolecular hydrogel system structures^46,47^. And each bio-ink exhibited porous microstructures with different chamber diameters, densities, and distributions. And the porosity of the scaffold was quantified by SEM of the scaffolds with imageJ software, as shown in Fig. 4g. The average porosity of the three scaffolds were 46.48%, 59.59% and 72.29%, respectively. With the increase of molecular weight distribution, the hole wall becomes thinner (Fig. S1). As previously reported in the literature, the porous structure of these hydrogels suggests their potential as scaffolds for cell infiltration, growth, and migration^26^.

### Mechanical properties

The mechanical properties of different 3D printing gelatin/alginate hydrogels with different heating–cooling times were also studied. Strain–stress curves of 3D printed scaffolds are shown in Fig. 4h. All scaffolds demonstrated good elasticity, with strains all greater than 80%. The fracture strain of scaffolds tended to increase with decreased heating–cooling times. As depicted in Fig. 4i, with increasing heating–cooling times, Young's modulus was getting weakened. The fracture stress of SA/G-A, SA/G-B, and SA/G-C scaffolds were 0.08962, 0.08392, and 0.0524 MPa, respectively. A decrease in mechanical properties results from higher porosity and lower crosslink density, which were caused by molecular structure^46^. The difference in molecular structure is the size and distribution of molecular weight, which is based on GPC data and FTIR spectra. Thus, molecular weight is the fundamental reason that affects the mechanical properties of scaffolds.

Studies have shown clear correlations between elastic moduli of hydrogel matrices and proliferation as well as differentiation of encapsulated cells^43^. Compared to the results of SA/G-A and SA/G-B, significantly lower stiffness values were achieved in SA/B-C despite the same composition (sodium alginate and gelatin hydrogels), the same crosslinking agent as well as the same crosslinking time. Besides, the material preparation method leads to lower molecular weight and increasing molecular weight distribution according to the analysis in section "Materials and methods"^43^. In conclusion, the method of heating–cooling decreases the mechanical properties, while leading to super shape fidelity and bioactivity.

### In vitro cytocompatibility after bioprinting

**Figure 5:**
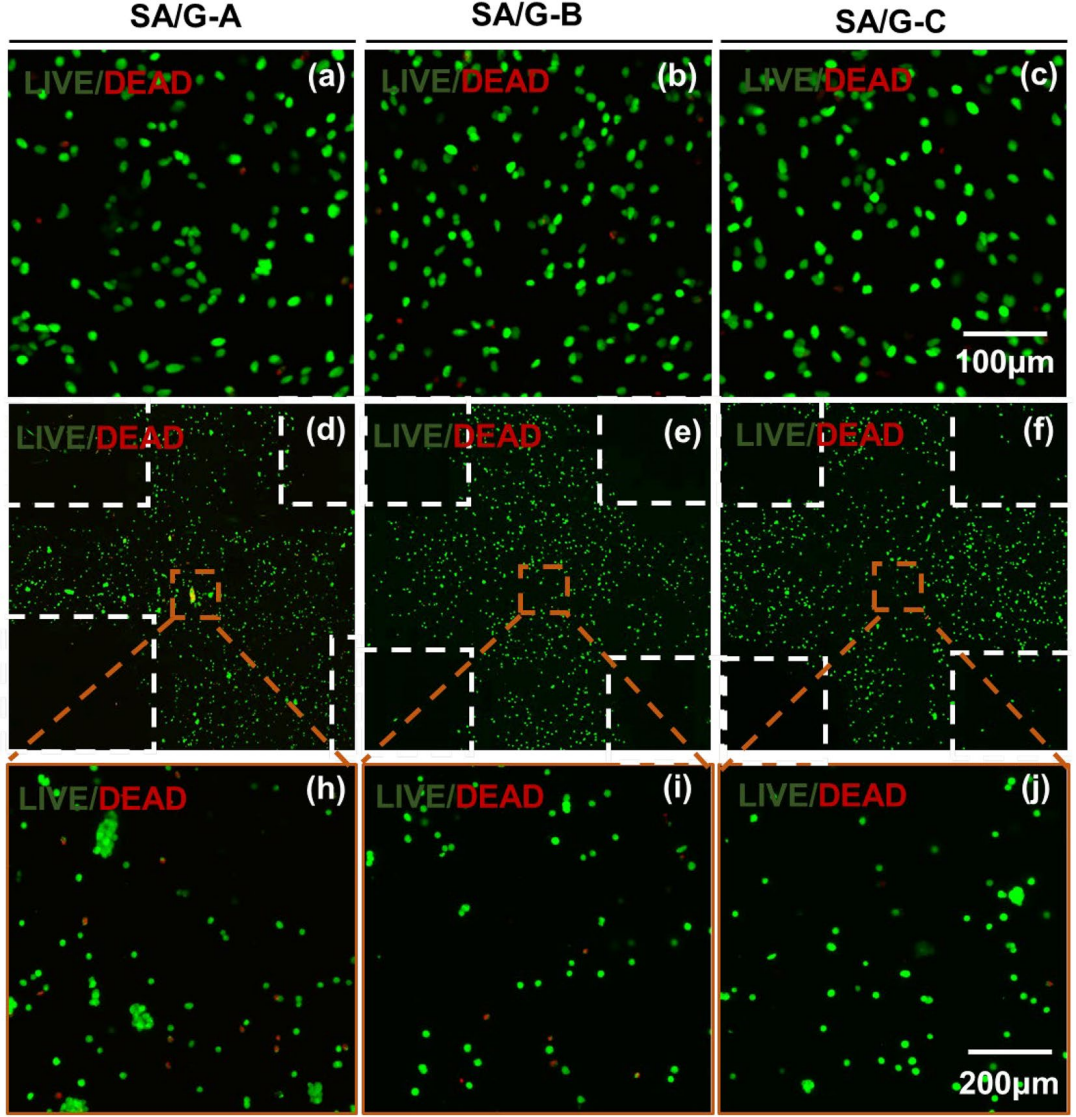
Cell distribution analysis. LIVE-DEAD staining cells before printing in (**a**) SA/G-A, (**b**) SA/G-B, and (**c**) SA/G-C hydrogel; (**d**)–(**f**) The cell LIVE/DEAD staining of (**a**), (**b**) after printing, respectively.

Since cell activity directly affects the proliferation, differentiation, and protein expression ability of scaffold cells, it is important to study the effect of cell survival during and after printing^25^. To determine the effects of bio-inks on cell behavior, the cell distribution, cell survival, and migration for 1 week were analyzed. Figure 5 shows the cell live/dead staining of SA/G bio-inks before printing and after printing. Before and after printing, the distribution trend of cells in semi-IPN bio-ink is the same. The distribution of cells in SA/G-A was the most uneven, which is far worse than that in SA/G-B and SA/G-C. This phenomenon is related to the viscosity of printing materials^34,35,43^. Nevertheless, the distribution of cells after printing is relatively more uneven. High viscosity materials are easy to block the nozzle, resulting in nonuniform extrusion of materials^25^. As the initial distribution of cells in construct made of SA/G-A is not uniform, the cells also showed uneven proliferation and diffusion.

**Figure 6:**
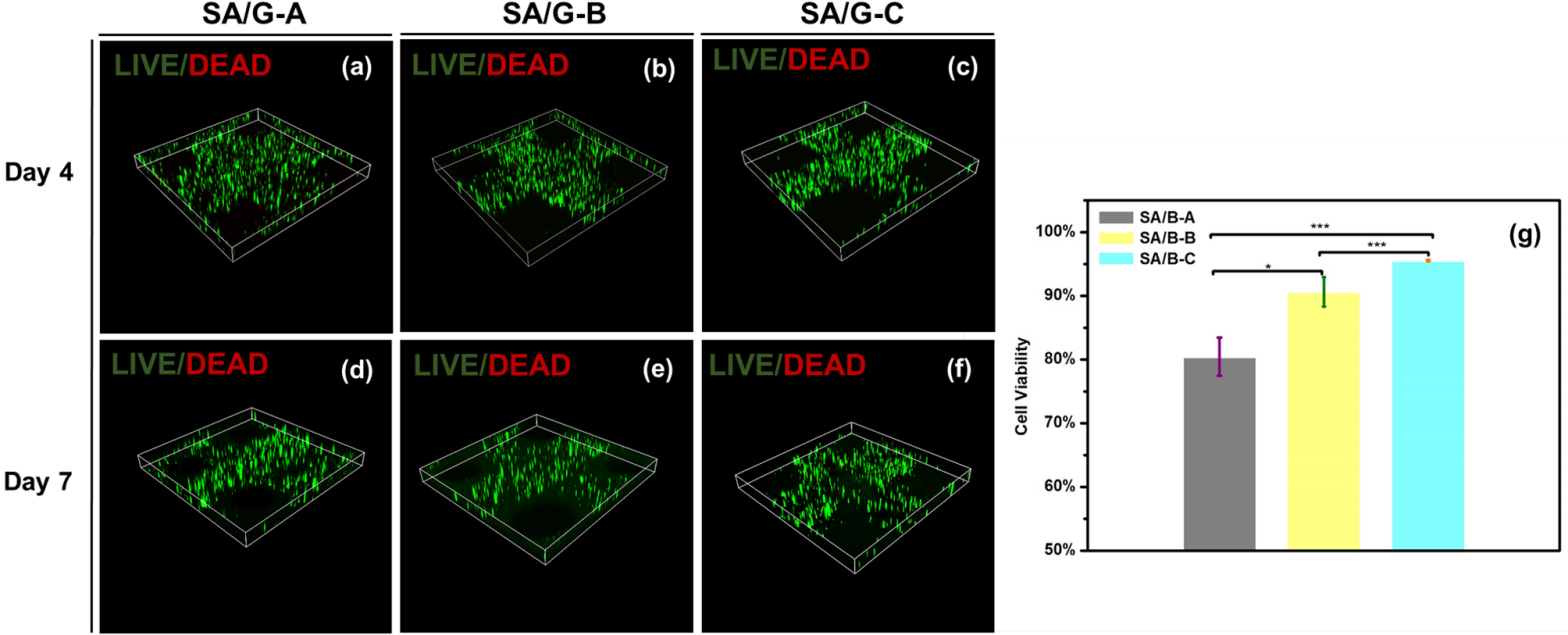
*C*ytocompatibility of bioprinted semi-IPN bio-ink. (**a**–**f**) A 3D view of the LIVE/DEAD staining on day4 and day7 after printing. (**g**) Cell viability on the day 1 after printing. All data are displayed as mean ± SD. ***indicates *p* < 0.00001, **indicates *p* < 0.0001, *indicates *p* < 0.005, analysed via Welch's *t* test.

Cytocompatibility of bioprinted semi-IPN bio-ink-containing cells was shown in Fig. 6. Cell survival rate at day1 increased significantly with increasing viscosity-shear sensitivity of bio-ink. The chondrocyte survival rates in the scaffolds made of SA/G-A, SA/G-B, and SA/G-C bio-inks were around 80%, 88%, and 95%, respectively (Fig. 6g). Indeed, fewer dead cells were observed in SA/G-C (Fig. 5f, i) compared with others. It confirmed our hypothesis that semi-IPN bio-ink with increasing molecular weight distribution can protect cells from stress and mechanical damage during the extrusion process^20,21,25,27^, resulting from which semi-IPN bio-inks with increasing molecular weight distribution is sensitive to shear stress^17,25^. SA/G-C can not only protect cells from mechanical pressure but also improve the printing accuracy.

In addition, A 3D view of the LIVE/DEAD staining on day4 and day7 after printing in Fig. 6a–f. A three-dimensional diagram showed that structures were “hollow”^52^ at day 7, especially Fig. 6f. The matrix strength of SA/G-C may be contributed to cell migration. The scaffold has appropriate matrix stiffness and high porosity, which is conducive to cell migration. The wide molecular weight distribution and low molecular weight resulted in a highly connected pore structure and appropriate matrix strength of SA/G-C^31^. This may be related to the loosening of the hydrogel network caused by the breakage of molecular chains. Recent studies have shown that the transport network is conducive to cell migration^47^.

**Figure 7:**
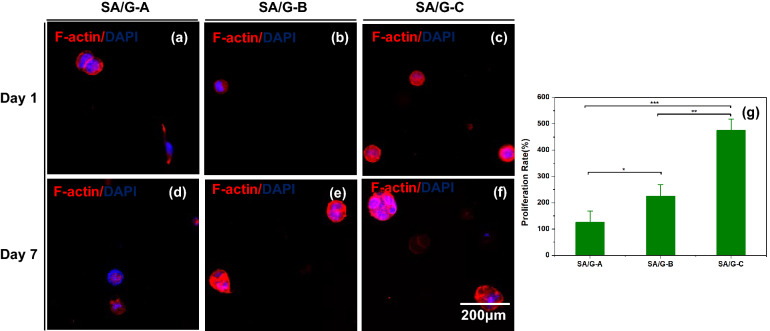
Cytoskeleton staining of the cell-laden scaffolds. Cytoskeleton staining view on day 1 (**a**–**c**) and on day7 (**d**–**f**). (**g**) Cell proliferation rate on day 7 after printing. All data are displayed as mean ± SD. ***indicates *p* < 0.0001, **indicates *p* < 0.001, *indicates *p* < 0.05, analysed via Welch's *t*-test.

F-actin (red) and DAPI (blue) staining were used to evaluate the cell spreading and proliferation within the bioprinted constructs (Fig. 7). The cells diffused in situ and proliferated into spherical aggregates in the 3D bioprinted constructs at day 7 post-printing. As noted in Fig. 7, one cell in SA/G-C proliferated to four to seven cells after 7 days of culture, and there was no obvious proliferation for cells in SA/G-A. According to the staining data and quantitative analysis by image J, the cell proliferation rates in the three materials were 125%, 225%, and 475%, respectively (Fig. 7g). Cells in SA/G-C bio-ink showed fast proliferation. Broken molecules that are not involved in the cross-linking process dissolve, making room for cell proliferation^34,35,43^.

Overall, SA/G-C bio-ink with a wide molecular weight distribution by cyclic heating–cooling treatment was prepared. And the relative molecular mass of this bio-ink became smaller, promoting a uniform cell distribution of cells; This semi-IPN bio-ink is more shear sensitive and can reduce the damage caused by the extrusion process and its interconnected porous structure provides space for cell proliferation^34,35,43^.

## Conclusion

To improve the printing accuracy and the cell survival rate after extrusion 3D printing, a method for developing cell-loaded semi-IPN bio-ink with high shear sensitivity was proposed in this study. Firstly, the recombination and reconstruction of molecular chains are realized by controlling the number of heating–cooling cycles and changing the length and flexibility of long molecular weight chains. The change of molecular-level changes the physicochemical properties of biological ink. This strategy prepared high shear thinning bio-ink, which not only improved cell survival and cell distribution but also improved cell proliferation. It is of great significance for 3D printing to guide the loaden cell print from the molecular structure of materials. Although bio-ink improves the printing accuracy and cell activity, cell differentiation, protein expression, and cartilage repair still need to be further studied. In addition, the methods and principles used in this study have guiding significance for the research of three-dimensional bioprinting polymer based on extrusion and are conducive to the development of tissue regeneration.

## Supplementary Information

Supplementary Figure S1.
