# Supplementary figures and images for: High-cytocompatible semi-IPN bio-ink with wide molecular weight distribution for extrusion 3D bioprinting
Source: Sci Rep. 2022 Apr 15;12:6349. doi: 10.1038/s41598-022-10338-1 (PMC9012805; doi:10.1038/s41598-022-10338-1)

**Supplement Information**


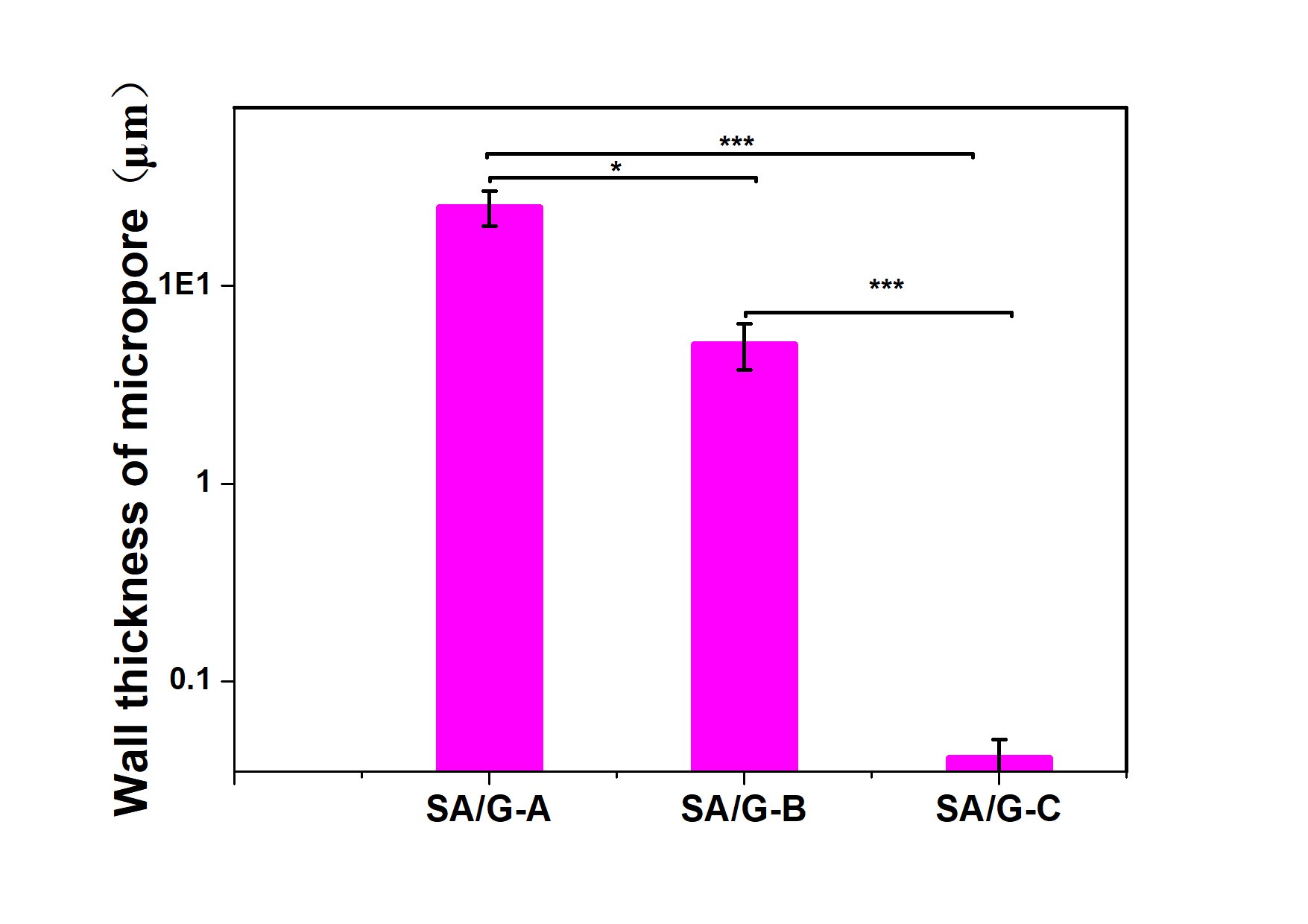


Figure.1 The wall thickness of micropore

Supplement: Supplementary file 1 — Supplementary Figure S1. [file 41598_2022_10338_MOESM1_ESM.docx]
